# Subset selection for domain adaptive pre-training of language model

**DOI:** 10.1038/s41598-025-94085-z

**Published:** 2025-03-19

**Authors:** JunHa Hwang, SeungDong Lee, HaNeul Kim, Young-Seob Jeong

**Affiliations:** Department of Computer Engineering, Chungbuk National University, Cheongju, 28644 Republic of Korea

**Keywords:** Computer science, Information technology

## Abstract

Pre-trained language models have brought significant performance improvements in many natural language understanding tasks. Domain-adaptive language models, which are trained with a specific domain corpus, exhibit high performance in their target domains. However, pre-training these models with a large amount of domain-specific data requires a substantial computational budget and resources, necessitating the development of efficient pre-training methods. In this paper, we propose a novel subset selection method called AlignSet, which extracts an informative subset from a given domain dataset for efficient pre-training. Our goal is to extract an informative subset that enables faster learning of the language model compared to learning from the entire dataset. By experiments across multiple domains, we demonstrate that AlignSet generates better subsets than other methods.

## Introduction

**Fig. 1:**
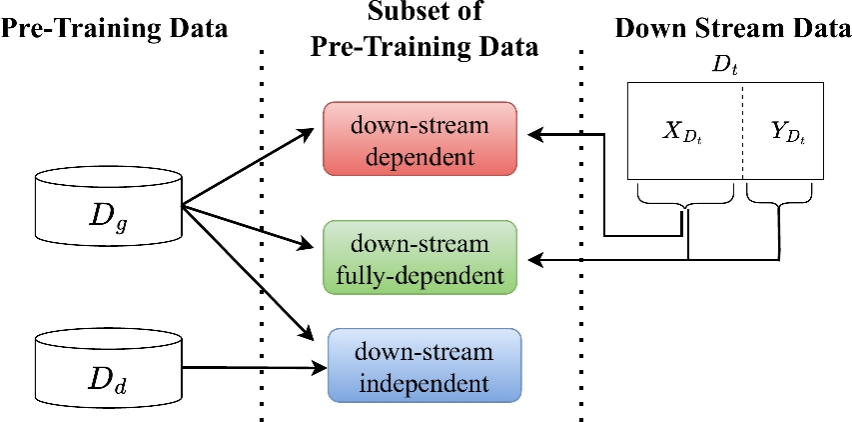
Three groups of subset selection methods according to the dependency to the down-stream dataset, where $D_g$ represents the pre-training corpus of the general domain, $D_d$ indicates the pre-training corpus of a target domain, and $X_{D_t}$ and $Y_{D_t}$ represent text and label in down-stream task dataset $D_t$, respectively.

With the advent of Transformer ^1^, pre-trained language models (PLMs) showed remarkable performance across a range of natural language understanding (NLU) tasks ^2–5^. Especially, domain-adaptive LMs obtained through continual pre-training (CPT) were found to be effective for target domains ^6–8^. Despite its effectiveness, the high cost of pre-training limits its applicability. For example, pre-training RoBERTa-large takes approximately one day using 1,024 V100 GPUs ^3^. Therefore, it is necessary to develop an efficient way to develop domain-adaptive LMs.

There are mainly two approaches for efficiently pre-training domain-adaptive LMs with a limited computational budget: a model-centric and a data-centric. The model-centric approach involves designing a compact model such as ALBERT ^9^ and EarlyBERT ^10^, whereas the data-centric approach focuses on data management or selection. As one of the data-centric approaches, subset selection refers to extracting only a portion of a large dataset that includes representative or informative instances, thereby creating a subset that is much smaller in size than the original dataset. Recently, there have been studies that efficiently train language models using subsets while maintaining or improving performance ^11–13^. However, these studies have limitations, such as relying on downstream task datasets or not outperforming random sampling.

The subset selection methods can be categorized into three groups based on the data dependency: *down-stream dependent*, *down-stream fully-dependent*, and *down-stream independent group*, as depicted in Fig. 1. The three groups commonly utilize a large general pre-training corpus $D_g$ that is used to pre-train general-purposed foundation models (e.g., Bidirectional encoder representations from transformers (BERT) ^2^). The upper two groups in the figure exploit the down-stream dataset $D_t$=$\{X_{D_t}, Y_{D_t}\}$ where $X_{D_t}$ and $Y_{D_t}$ indicate the input texts and the output labels, respectively. Specifically, the *down-stream dependent* group takes $D_g$ and $X_{D_t}$, and the *down-stream fully-dependent* group employs $D_g$, $X_{D_t}$ and $Y_{D_t}$. The dependency to the down-stream dataset $D_t$ may cause bias when we do not have a sufficient amount of the down-stream data. On the other hand, the *down-stream independent* group does not suffer from this issue as it takes only $D_g$ and $D_d$, where $D_d$ is a dataset of a target domain. Note that the $D_d$ is unlabelled corpus in the target domain, whereas the general dataset $D_g$ incorporates multiple domains.

In this study, we propose a new subset selection method called *AlignSet*, belonging to the *down-stream independent group*, to efficiently pre-train a domain-adapted LM within a limited computational budget while avoiding the aforementioned limitations. The main idea of AlignSet is to align sentence representations obtained from two different PLMs so that we can exploit the aligned representations to score examples. Based on the finding that there exist representative subsets for a target domain ^13^, our goal is to extract an informative subset that makes the language model better work on domain-specific tasks. Note that our model is designed to extract a subset in such a way that the language model achieves better performance on target domain tasks compared to a randomly selected subset. However, it is not designed to outperform a language model trained on the entire dataset. Our contributions are as follows.

- AlignSet: We propose a new subset selection method of *down-stream independent* group. It utilizes the similarity between aligned embeddings obtained from two PLMs.
- Experiments on multiple domains: Similar to Gururangan et al. ^14^, we perform the experiments on several domains (e.g., biomedical science, computer science, news, and personality), and show that the proposed method outperforms other subset selection methods.

## Related works

### Pre-training language models

After Transformer ^1^ sheds light on natural language processing (NLP) field, language models (LMs) using Transformer architecture pre-trained on large datasets from the general domain have shown outstanding performance across various NLP tasks. ^2,4,15,16^ BERT ^2^, one of the most well-known LMs, employs the encoder part of the Transformer, effectively capturing latent representations and making it applicable to various NLU tasks. Such encoder-based LMs are known as powerful to NLU tasks as it is designed to encode or comprehend the context of given texts. Indeed, compared to decoder-based LMs (e.g., Generative Pre-trained Transformer (GPT) series ^4,15,17,18^, Llama series ^19–21^), the encoder-based LMs mostly achieve the better performance on NLU tasks if the LMs are of the same scale (i.e., same model size). Furthermore, recent studies found that adapting LMs to a target domain through fine-tuning or continual pre-training (CPT) contributes to performance gain. In this paper, we focus on the encoder-based LMs and propose a new subset selection method for efficient CPT.

### Efficient pre-training

While the pre-training ^22,23^ or continual pre-training(CPT) ^14^ is a promising way to build a general or domain-adaptive language model, it demands significant computational resources. ^24^ To mitigate this issue, there have been studies on model-centric and data-centric approaches to efficiently pre-train language models with a limited computational budget. As a model-centric approach, Chen et al. ^10^ designed a way of training models based on the lottery ticket hypothesis. Lan et al. ^9^ and Dehghani et al. ^25^ introduced parameter sharing techniques for making the models to have fewer parameters. Such parameter sharing enables better performance in language modeling and allows to efficiently construct the language models. In this paper, we chose the ALBERT ^9^ utilizing such efficient parameter sharing technique as our base model for experiments.

As a data-centric approach, there have been subset selection techniques that extract informative examples from a large dataset to construct a small but effective or representative subset. The subset allows efficient training without losing much performance compared to the total dataset. Yao et al. ^11^ employed BM25^26^ by treating the textual data of down-stream task as a query. This allows the similarity measurement between the pre-training data and the query to facilitate subset selection. Wang et al. ^12^ introduced a subset selection based on influence function ^27,28^, and showed that their method outperforms other previous domain-specific language models. It finds samples that have the most positive impact on the down-stream task using the influence function. These studies commonly rely on the dataset of target task $D_t$; they are of *down-stream dependent* or *down-stream fully-dependent* groups. Suzuki et al. ^13^, belonging to *down-stream independent* group, defined scores based on the difference in length-normalized per-word cross-entropy between a domain-adaptive language model $M_d$ and a general language model $M_g$. They proved that there exist representative subsets for a given domain, but their method did not exhibit much performance improvement over randomly extracted subsets. We believe that the biggest reason for their marginal performance gain might be that they strongly rely on the model loss, so it became difficult to distinguish between noisy samples and informative samples ^29,30^.

**Fig. 2:**
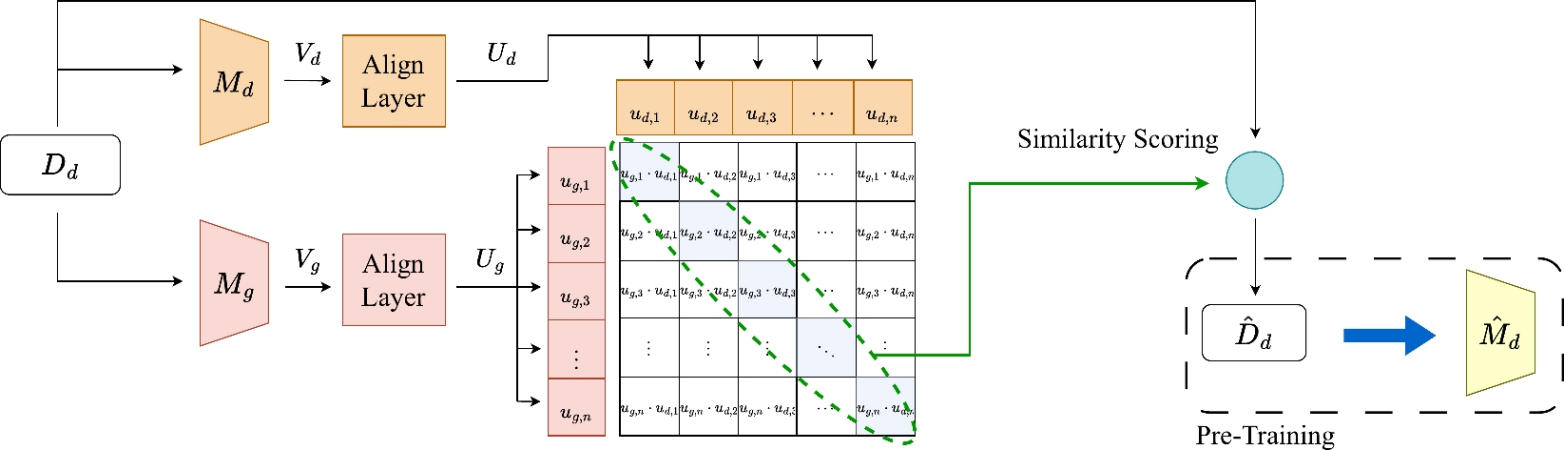
The overview of AlignSet, where $\hat{D}_d$ is a subset of $D_d$, $\hat{M}_d$ is a domain-adaptive language model, $V_d$ and $V_g$ are embeddings generated by $M_d$ and $M_g$, respectively. And $U_d$ and $U_g$ refer to the embeddings projected into the unified embedding space via the alignment layer.

**Table 1:** Data specification of the domain-specific datasets $D_d$ and down-stream datasets $D_t$ across multiple domains.

Purpose	Domain	Dataset	Task	Subset ratio	Size		
					Train	Val	Test
pre-training($D_d$)	CS, BIOMED	S2ORC ^31^	–	10%	10,610,430	–	- -
	News	CCNEWS ^32^	–	27%	708,241	–	–
	Personality	Pandora ^33^	–	10%	17,640,062	–	–
down-stream($D_t$)	CS	ACL-ARC ^34^	Citation intent classification	–	1688	114	139
	BIOMED	RCT ^35^	Abstract sentence roles classification	–	180,040	30,212	30,135
	News	AGNEWS ^36^	Topic classification	–	115,000	5000	7600
		HYPERPARTISAN ^37^	Partisanship classification	–	514	63	65
	Personality	First impressions V2 ^38^	OCEAN factor regression	–	6000	2000	2000
	Review	HELPFULNESS ^39^	Helpfulness classification	–	115,251	5000	25,000
		IMDB ^40^	Sentiment classification	–	20,000	5000	25,000

## Method

Suppose we want to construct a domain-adaptive language model for a target domain *d*. The best way is to borrow a general-purposed language model (i.e., a language model pre-trained with general domains), and adapt it to the domain *d* through fine-tuning or continual pre-training (CPT). However, even if we have a pre-training corpus $D_d$ of the domain *d*, it is expensive to perform CPT with the entire corpus $D_d$. Our subset selection method extracts a subset $\hat{D}_d$ that comprises a certain portion of $D_d$, where the subset $\hat{D}_d$ is much smaller than $D_d$ but contains informative samples. In this paper, we arbitrarily set $|\hat{D}_d| \approx (0.1 \sim 0.27) \times |D_d|$ based on our limited computation resources. Please refer to the subsection of Experimental settings and datasets for more details.

### AlignSet

Following Suzuki et al. ^13^, we assume that we have two pre-trained language models (PLMs): a general-purposed language model $M_g$ and an initial in-domain language model $M_d$ of the target domain, as shown in Fig. 2. Any model can be chosen as the initial model $M_d$ when it is pre-trained with any small amount of the target domain; in this paper, as random selection is the simplest way to get a subset, a language model pre-trained with a randomly selected subset of the target domain is employed as $M_d$. The goal of this study is to design a subset selection method for efficiently pre-training language models, so it might seem problematic that the initial model is already a PLM. It is worth noting that the initial model is pre-trained with an arbitrary in-domain subset (e.g., random subset), and we use such initial model as a proxy for the target domain to extract an *informative* subset that provides performance gain. Through experiments, we demonstrate that AlignSet is effective and efficient, even when considering the time required to build the initial model.

The two PLMs $M_d$ and $M_g$ take the same set of texts as input and yield sentence embeddings $V_d \in \mathbb {R}^{N \times m_{d}}$ and $V_g \in \mathbb {R}^{N \times m_{g}}$, where *N* is the number of sentences in a batch. The embeddings can be any vectors derived from the PLMs (e.g., output vectors of [CLS] token, mean vectors of all tokens, etc). The two PLMs have different embedding spaces, so $V_d$ and $V_g$ must be different even for the same text. Inspired by Radford et al. ^41^ and Karpukhin et al. ^42^, we design *align-layer* that projects $V_d$ and $V_g$ into an unified embedding space, where the projected embeddings $U_d \in \mathbb {R}^{N \times m}$ and $U_g \in \mathbb {R}^{N \times m}$. The unified embedding space allows to get the similarity between sentence embeddings, where the diagonal values are self-similarities, as depicted in Fig. 2.

The motivation of AlignSet is as follows. First, we assume that if a particular example differs from or is more distinguishable than the others, then the example is more informative. To find such informative examples, we introduce a contrastive loss that measures the similarity of an example to itself and to other examples. This approach enables us to train a unified embedding space that captures the similarity between the two PLMs. After training with contrastive loss, a high similarity between positive pairs indicates that they are clearly distinguishable from other examples. In other words, examples with high similarity across the two PLMs are considered more distinctive or informative compared to others. Second, if we simply apply the contrastive loss to a target domain using a single model (i.e., $M_g$=$M_d$), it may lead to selecting only abnormal examples or outliers, as is often the case in the computer vision area ^43,44^. Therefore, we design the aligned space by introducing $M_g$ and $M_d$, where $M_g$ has a base or general perspective (i.e., distribution) and $M_d$ has a perspective of the target domain. We expect that the aligned space between the two PLMs will allow to find domain-informative examples that are not merely abnormal or outliers.

### Loss function

Following Radford et al. ^41^, we utilize the InfoNCE loss function ^45^ to train the align-layer parameters:

1 $$\begin{aligned} L_{NCE}(v_i, W) = -log \frac{e^{sim(v_i, w_i) / \tau }}{\sum _{w_j \in W} e^{sim(v_i, w_j) / \tau }} \end{aligned}$$

where $\tau$ is a temperature, $w_i$ and $v_i$ indicate *i*-th element of embeddings generated by the two language models within the batch, and *W* = $\{w_1,..., w_{|W|}\}$. The temperature parameter controls the range of the logits in the softmax function and is a trainable parameter that is directly optimized during training. In this paper, we employ cosine similarity as the similarity function *sim* between embeddings. We apply this loss both column-wise and row-wise, and obtain the total loss *L* as below.

2 $$\begin{aligned} L = (L_{NCE}(u_{g, i}, U_d) + L_{NCE}(u_{d, i}, U_g)) / 2 \end{aligned}$$

The loss encourages maximizing the similarity of *positive pairs* while minimizing the similarity of *negative pairs*. In other words, positive pairs are pulled closer together, while negative pairs are pushed farther apart in aligned space. The positive pairs, represented by the diagonals in Fig. 2, correspond to the embeddings of the same examples, whereas the negative pairs correspond to the embeddings of different examples. During the training phase, $M_g$ and $M_d$ are frozen, and the parameters of the align-layer are updated. After training, the positive pairs are used to compute the self-similarity scores. Specifically, self-similarity scores for positive pairs can be obtained from the trained model’s unified embedding space as an inference result. We utilize this score to measure the informativeness of each example, and the top-*k* examples are selected as the subset $\hat{D}_d$.

## Experiments

**Table 2:** Averaged results with standard deviations on four domains, where $d_1 \rightarrow d_2$ indicates the domain adaptation from $d_1$ (domain of $D_d$) to $d_2$ (domain of $D_t$).

Domain adaptation	Task dataset	Vanilla model (w/o further pre-training)	RandomSet	SuzukiSet	AlignSet	FullSet
BioMed $\rightarrow$ BioMed	RCT	85.71 ± 0.06	86.47 ± 0.08	86.39 ± 0.08	**86.52 **± **0.04**	-
CS $\rightarrow$ CS	ACL-ARC	74.07 ± 5.60	75.71 ± 2.01	75.56 ± 2.48	**76.15 **± **1.51**	-
News $\rightarrow$ News	AGNEWS	93.73 ± 0.19	93.90 ± 0.20	93.99 ± 0.06	**94.12 **± **0.13**	93.84 ± 0.13
	HYPERPARTISAN	81.81 ± 4.67	84.84 ± 4.35	84.95 ± 1.26	**86.45 **± **1.98**	82.63 ± 2.10
Personality $\rightarrow$ Personality	First Impressions V2	0.1128 ± 0.0013	0.1120 ± 0.0004	0.1121 ± 0.0004	**0.1118 **± **0.0001**	–
$\dagger$ Personality $\rightarrow$ Review	HELPFULNESS	68.40 ± 0.65	68.21 ± 0.39	68.19 ± 0.28	**68.42 **± **0.46**	–
	IMDB	**93.64 **± **0.01**	93.32 ± 0.20	90.95 ± 0.24	93.35 ± 0.04	–

We used macro-F1 for the classification tasks, but micro-F1 for the RCT dataset. Mean absolute error (MAE) was utilized for the First Impressions V2 dataset. $\dagger$ indicates cross-domain adaptation.

Significant values are in bold.

### Experimental settings and datasets

For our limited computational resource, we chose to use A Lite BERT (ALBERT) ^9^ for all models (e.g., $M_g$, $M_d$, and $\hat{M}_d$). Our method only learns the aligned space between encoder-based models without modifying architecture, making it easily applicable to other encoder-based models. We utilized a publicly available general-purposed ALBERT checkpoint from HuggingFace as $M_g$. As explained earlier, any model can be chosen as $M_d$ if it is pre-trained with a small subset of the target domain. In our experiments, $M_d$ is obtained by performing continual pre-training (CPT) on $M_g$ with a randomly selected subset of $D_d$. For AlignSet, we set the [CLS] token representations of the last layer as $V_g$ and $V_d$, and employ a linear layer as the align-layer, which is randomly initialized. When we fine-tune the model and predict task labels, the representation of the [CLS] token is passed to the task-specific linear layer.

We conducted experiments on multiple domains: computer science (CS), biomedical science (BioMed), news, personality, and review, where the review domain is for cross-domain experiments. Table 1 summarizes the source and specification of the in-domain pre-training datasets $D_d$ and down-stream task datasets $D_t$, where all datasets are in English. The subset ratio (i.e., 10$\sim$27%) is chosen based on the amount of time required to pre-train the models because it mostly takes too long for pre-training. For example, in the personality domain, it took 358 hours (15 days) for a single run of pre-training with the 10% subset. On the other hand, the dataset size of the news domain is much smaller than the other domains, so we arbitrarily set the subset size to be 10% of the personality domain subset, which corresponds to 27%. The task of $D_t$ is a classification task, except for the First Impressions V2 dataset which is a regression task on OCEAN factors: openness, conscientiousness, extraversion, agreeableness, and neuroticism. Specifically, the OCEAN factor regression is to predict per-factor scores ranging from 0 to 1 for a given input text. Therefore, we took mean absolute error (MAE) as a metric for the OCEAN factor regression, while we report the classification results using macro-F1 and micro-F1 by following Gururangan et al. ^14^ and Beltagy et al. ^6^. All experimental results are averages of five independent runs with random seeds.

In the pre-training steps, we used Adam optimizer ^46^ with decoupled weight decay regularization ^47^, and the initial learning rate was 0.0001. We used the batch-size of 16, and the number of epochs was 20. We adopted a linear learning rate scheduler with 10,000 warm-up steps. In the fine-tuning steps, we used the batch-size of 32, and an initial learning rate of 0.00003 with 10 epochs. The experiments were conducted on 4 NVIDIA GeForce RTX 3090 GPUs.

## Results

### Main result

We compared the AlignSet with two rival methods: a randomly selected subset (RandomSet) and a subset obtained from the method of Suzuki et al. ^13^ (SuzukiSet). Table 2 summarizes the experimental results on multiple domains, where FullSet indicates the entire pre-training dataset $D_d$. We examined the FullSet only on the news domain due to our limited computational budget. Note that the domains of $\hat{D}_d$ and the task dataset are the same for BioMed, CS, news, and personality domains, whereas cross-domain adaptation was examined on the review domain. The vanilla model indicates the general purposed model $M_g$ without any continual pre-training. The subset selection methods generally outperformed the vanilla model, which is consistent with previous studies showing that domain-adaptive models are superior to general models for target domains. The AlignSet achieved the best performance amongst them, implying that the AlignSet finds better subsets using the aligned embedding space.

### Impact of in-domain pre-training dataset

Although the subset selection method is mainly for efficient pre-training, we examined the task performance of the FullSet on the news domain. We found that the RandomSet was comparable to the FullSet, but the other subset selection methods exhibited better performance than the FullSet. This result is associated with the size of $D_d$. As shown in Table 1, $D_{d}$ of the news domain is only 4$\sim$6% of other domains, causing a greater distribution shift during the subset selection. Specifically, we speculate that RandomSet performed better than FullSet because it coincidentally happened to be closer to the distribution of the fine-tuning dataset. The performance gap caused by the distribution shift will be relieved as the size of $D_d$ gets larger ^48,49^. We also conducted *cross-domain adaptation* (i.e., $personality \rightarrow review$) to check the importance of the in-domain pre-training dataset $D_d$ and how well our method works for unseen domains. As shown at the bottom of Table 2, the subset selection methods were comparable to or worse than the vanilla model because the domains of $D_d$ and $D_t$ are different. This implies that selecting an appropriate $D_d$ is crucial for performance.

### Efficiency

As the essential goal of the subset selection is efficient pre-training, we examined how long it takes to construct the subsets. Table 3 summarizes the elapsed time of different subset selection methods. The elapsed time for AlignSet includes align-layer training (e.g. 88 h) and data selection (e.g. 15 h). The RandomSet was the fastest because it just picks arbitrary examples. Even though the AlignSet takes longer than the SuzukiSet, the AlignSet is still more efficient than the FullSet. Assuming that we build a domain-adaptive LM for the personality domain. If we perform continual pre-training (CPT) for 20 epochs with FullSet, then it takes longer than 150 days using 4 NVIDIA GeForce RTX 3090 GPUs. On the other hand, the AlignSet will take only 34 days, which is almost one-fifth of the FullSet. That is, it takes 15 days to prepare the in-domain initial model $M_d$ (i.e., pre-training $M_d$ using the RandomSet), 103 hours (4.3 days) for subset selection, and another 15 days for pre-training the domain-adaptive LM $\hat{M}_d$. We also investigate the memory consumption efficiency of each subset selection method using Big-O notation. For RandomSet, since samples are randomly selected from the entire dataset, it requires *O*(1). On the other hand, SuzukiSet and AlignSet operate in small batches of size *B*, requiring $O(B^2)$. This memory consumption does not account for the complexity of model training and applies only to additional memory complexity.

### Aligned space

**Fig. 3:**
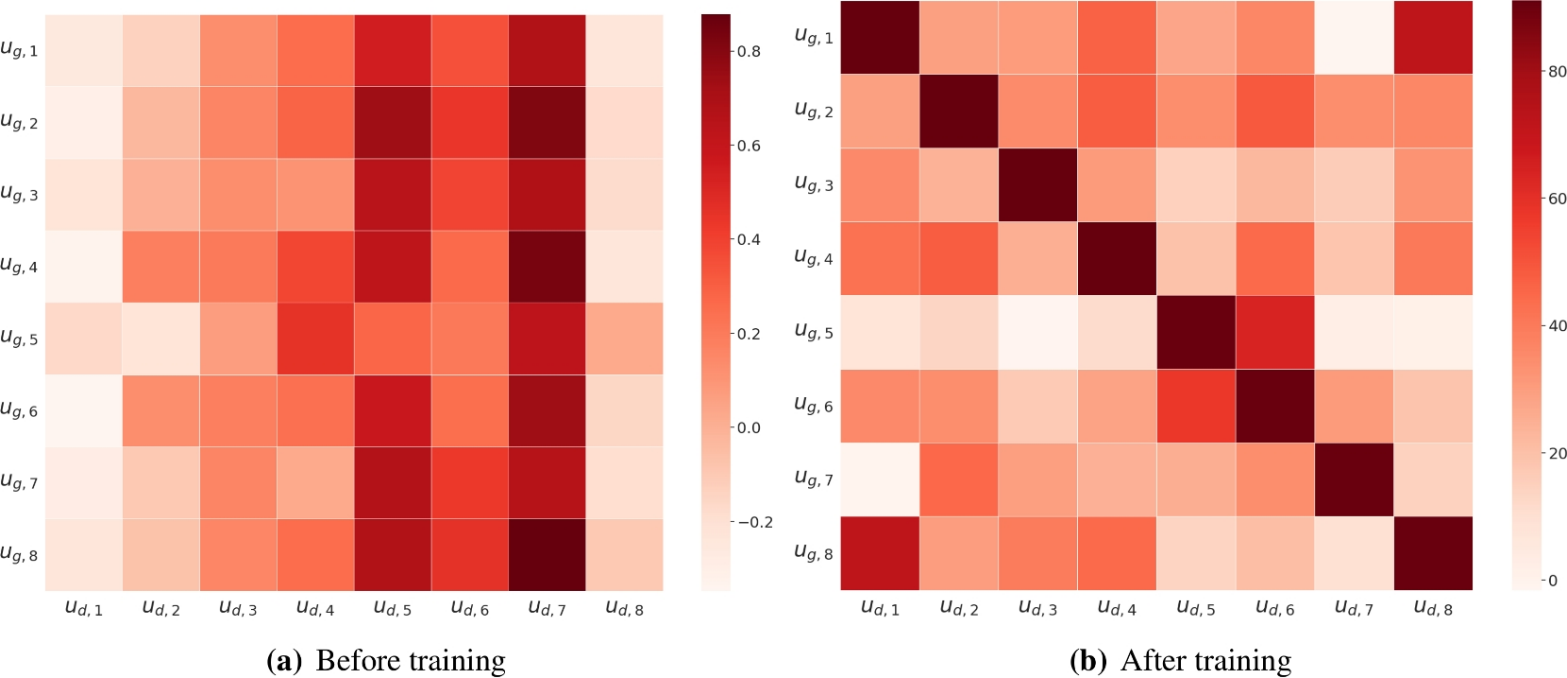
Heatmaps of aligned embedding space, where the columns and rows correspond to $U_d$ and $U_g$, respectively.

To check if the AlignSet forms the aligned embedding space well, we visualized the embedding space using arbitrary eight examples, as shown in Fig. 3 where the columns and rows of the heatmaps denote $U_d$ and $U_g$ within Fig. 2, respectively. In Fig. 3b, aligned diagonal values imply that the align-layer is well-trained and properly maps the sentence representations to the aligned space.

### Subset visualization

**Fig. 4:**
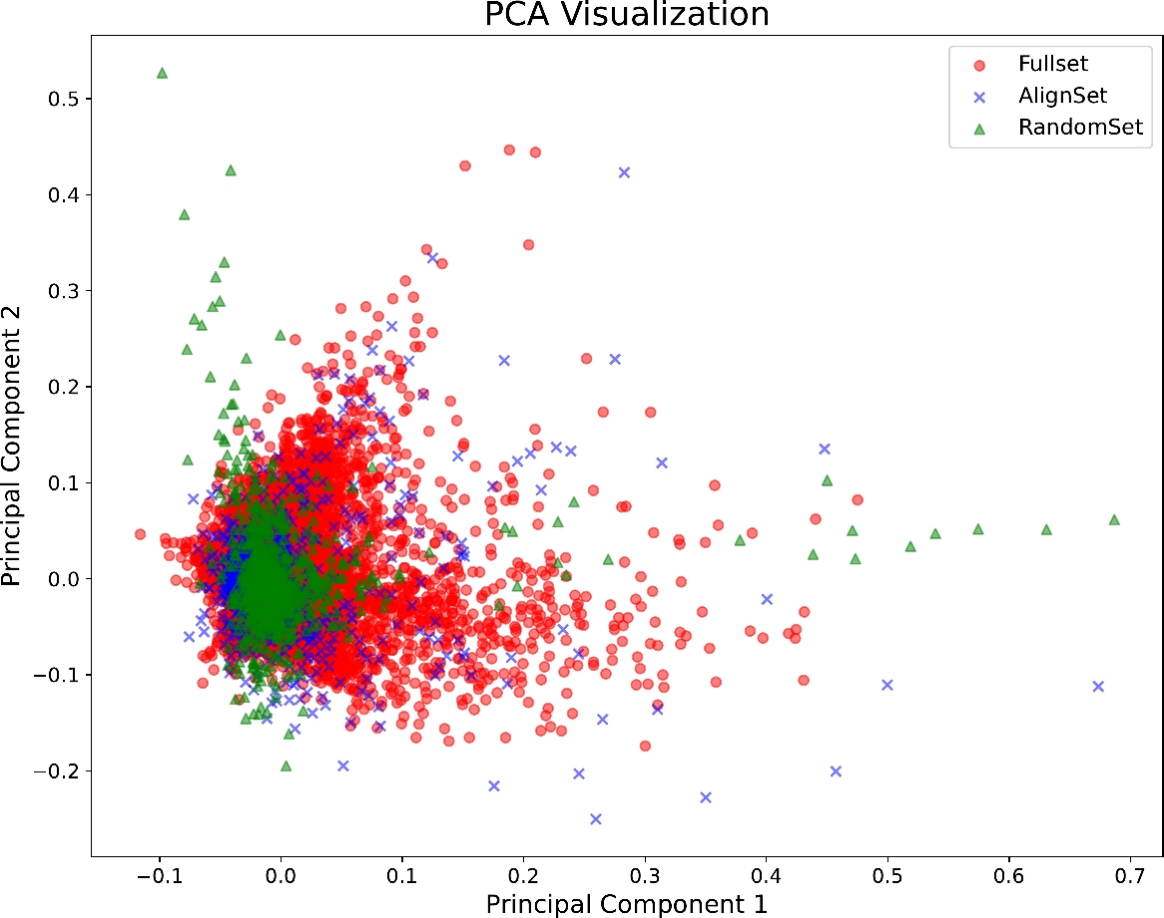
PCA visualization of FullSet, RandomSet, and AlignSet in the CS domain, where circles represent FullSet, and triangles and X markers indicate RandomSet and AlignSet, respectively.

To assess the quality of the subsets, we visualized AlignSet, RandomSet, and FullSet using PCA (principal component analysis) plots and presented in Fig. 4. For visualization, we randomly selected 10,000 samples from the FullSet and 1000 samples from each remaining subset in the CS domain. In the figure, FullSet is represented by red circles, RandomSet by green triangles, and AlignSet by blue X markers. AlignSet appears to better reflect the distribution of FullSet compared to RandomSet. Specifically, while RandomSet captures FullSet in a more localized manner, AlignSet preserves relatively more essential characteristics of FullSet.

**Table 3:** Elapsed time of subset selection methods.

Domain	RandomSet	SuzukiSet	AlignSet
CS, BioMed	4 s	31 h	74 h
News	1 s	2 h	6.5 h
Personality	4 s	74 h	103 h

## Limitation

This study has a limitation that we utilized the ALBERT checkpoint for all the experiments due to our limited computational budget; at the early stage of this study, we found that the estimated time for pre-training RoBERTa ^3^ or BigBird ^50^ with RandomSet of the personality domain takes longer than 50 days.

Another limitation of this paper is that we did not conduct experiments with decoder-based models (e.g., GPT ^4,51^ or Llama ^52,53^ series). Previous articles discovered the potential of domain-adaptive generative models ^54,55^, and we believe that the AlignSet will be able to contribute to the efficient development of domain-adaptive decoder-based models. The AlignSet is just a subset selection method, so it will be applicable to any other domains and multimodal tasks. However, the low-resource languages (LRL) have relatively small datasets, so dataset augmentation techniques will be more appropriate instead of subset selection methods.

Furthermore, we chose the subset size based on the time required to pre-train the models. Based on the previous analysis ^1^, the time complexity of training transformer is $O(N \cdot L^2 \cdot d)$, where *L* represents sequence length, *d* and *N* indicate dimension and data size, respectively. Since the actual training time is influenced by the hardware environment, we manually checked the training time on our machine and determined the subset ratio accordingly.

## Conclusion

To reduce pre-training costs for a target domain, we proposed a novel subset selection method AlignSet. The AlignSet is designed to align the sentence representations of two different PLMs by a unified embedding space, and examples with high self-similarity scores are selected as a subset. By experimental results on multiple domains, we showed that the AlignSet allows to efficiently pre-train domain-adaptive LMs. As on-device language models (i.e., small language models) are having attention lately due to the heavy computational cost of training language models, we believe that our subset selection method will contribute to developing small but effective language models for a target domain. We also plan to extend the AlignSet by applying it iteratively; when we get the domain-adaptive LM $\hat{M}_d$, then it can be used as the initial model $M_d$ for another round of AlignSet. We expect that through this process, the quality of AlignSet will improve. However, this will also lead to an increase in training time. In other words, such an iterative way may degrade in terms of efficiency, but we expect to find a suitable balance between efficiency and effectiveness.

## Data Availability

All datasets used in the current study, except for Pandora and First Impression V2, are publicly available: S2ORC (https://huggingface.co/datasets/allenai/peS2o), CCNEWS (https://huggingface.co/datasets/vblagoje/cc_news), others (https://github.com/allenai/dont-stop-pretraining). The Pandora and First Impressions V2 datasets are available upon reasonable request and with permission from each author: Pandora (https://psy.takelab.fer.hr/datasets/all/pandora/), First Impressions V2 (https://chalearnlap.cvc.uab.cat/dataset/24/description/).
